# Dyspnea is severe and associated with a higher intubation rate in de novo acute hypoxemic respiratory failure

**DOI:** 10.1186/s13054-024-04903-5

**Published:** 2024-05-23

**Authors:** Alexandre Demoule, Amandine Baptiste, Arnaud W. Thille, Thomas Similowski, Stephanie Ragot, Gwénael Prat, Alain Mercat, Christophe Girault, Guillaume Carteaux, Thierry Boulain, Sébastien Perbet, Maxens Decavèle, Lisa Belin, Jean-Pierre Frat, from the REVA Network (Research Network in Mechanical Ventilation)

**Affiliations:** 1 INSERM, UMRS1158 Neurophysiologie Respiratoire Expérimentale et Clinique, Sorbonne Université, 75005 Paris, France; 2 Groupe Hospitalier Universitaire APHP-Sorbonne Université, Site Pitié-Salpêtrière, Service de Médecine Intensive et Réanimation (Département R3S), Hôpital Universitaire Pitié-Salpêtrière, AP-HP, 47-83 Boulevard de L’Hôpital, 75651 Paris Cedex 13, France; 3 Groupe Hospitalier Universitaire APHP-Sorbonne Université, Site Pitié-Salpêtrière, Unité de Recherche Clinique, AP-HP, Paris, France; 4 Médecine Intensive Réanimation, Centre Hospitalier Universitaire de Poitiers, Poitiers, France; 5 Centre d’Investigation Clinique 1402 ALIVE, Université de Poitiers, Poitiers, France; 6 Groupe Hospitalier Universitaire APHP-Sorbonne Université, Site Pitié-Salpêtrière, Département R3S, AP-HP, 75013 Paris, France; 7 Service de Médecine Intensive et Réanimation, CHU de Brest, Brest, France; 8 Service de Réanimation médicale et Médecine Hyperbare, Centre Hospitalier Régional Universitaire, Angers, France; 9 UNIROUEN, UR 3830, Medical Intensive Care Unit, Rouen University Hospital, Normandie University, Rouen, France; 10 Hôpitaux Universitaires Henri Mondor, Service de Médecine Intensive Réanimation, Université Paris Est Créteil, Groupe de Recherche Clinique CARMAS, AP-HP, Créteil, France; 11 Médecine Intensive Réanimation, Centre Hospitalier Universitaire d’Orléans, Orléans, France; 12 Réanimation Médico-Chirurgicale, CHU de Clermont-Ferrand, Clermont-Ferrand, France; 13 GReD, UMR/CNRS 6293, INSERM U1103, Université Clermont Auvergne, Clermont-Ferrand, France; 14 Site Pitié-Salpêtrière, Département de Santé Publique, INSERM, Institut Pierre Louis d’Epidémiologie Et de Santé Publique, AP-HP, APHP-Sorbonne Université, Paris, France

**Keywords:** Dyspnea, High-flow oxygen therapy, Mortality, Intubation, Acute respiratory failure

## Abstract

**Background:**

Dyspnea is a key symptom of de novo acute hypoxemic respiratory failure. This study explores dyspnea and its association with intubation and mortality in this population.

**Methods:**

This was a secondary analysis of a multicenter, randomized, controlled trial. Dyspnea was quantified by a visual analog scale (dyspnea-VAS) from zero to 100 mm. Dyspnea was measured in 259 of the 310 patients included. Factors associated with intubation were assessed with a competing risks model taking into account ICU discharge. The Cox model was used to evaluate factors associated with 90-day mortality.

**Results:**

At baseline (randomization in the parent trial), median dyspnea-VAS was 46 (interquartile range, 16–65) mm and was ≥ 40 mm in 146 patients (56%). The intubation rate was 45%. Baseline variables independently associated with intubation were moderate (dyspnea-VAS 40–64 mm) and severe (dyspnea-VAS ≥ 65 mm) dyspnea at baseline (sHR 1.96 and 2.61, *p* = 0.023), systolic arterial pressure (sHR 2.56, *p* < 0.001), heart rate (sHR 1.94, *p* = 0.02) and PaO_2_/FiO_2_ (sHR 0.34, *p* = 0.028). 90-day mortality was 20%. The cumulative probability of survival was lower in patients with baseline dyspnea-VAS ≥ 40 mm (logrank test, *p* = 0.049). Variables independently associated with mortality were SAPS 2 ≥ 25 (*p* < 0.001), moderate-to-severe dyspnea at baseline (*p* = 0.073), PaO_2_/FiO_2_ (*p* = 0.118), and treatment arm (*p* = 0.046).

**Conclusions:**

In patients admitted to the ICU for de novo acute hypoxemic respiratory failure, dyspnea is associated with a higher risk of intubation and with a higher mortality.

**Trial registration**: clinicaltrials.gov Identifier # NCT 01320384.

**Supplementary Information:**

The online version contains supplementary material available at 10.1186/s13054-024-04903-5.

## Introduction

Acute respiratory failure (ARF) results from an imbalance between the load and the capacity of the respiratory system. Noninvasive respiratory support such as high flow oxygen therapy (HFOT) and noninvasive ventilation (NIV) may prevent intubation by reducing load capacity imbalance and hence signs of respiratory distress [1, 2]. Early prediction of the need for intubation in patients with de novo hypoxemic ARF remains challenging. Many factors supposed to predict intubation, such as severe hypoxemia, shock or coma, are themselves actually intubation criteria [3–7]. The best prediction accuracy of the ratio of respiratory-rate-oxygenation index is after 12 h of HFOT [8]. Tidal volume is also a good predictor of intubation, but only in patients receiving NIV [5, 9, 10].

Dyspnea, the abnormal and distressing awareness of breathing, is a key symptom of ARF [11]. As opposed to physical signs of respiratory distress such as tachypnea and labored breathing, dyspnea is a symptom, which places a very strong emphasis on self-reporting [11]. Because it parallels respiratory drive, dyspnea is a marker of load capacity imbalance [12]. As such, dyspnea could help to predict intubation. Reports on dyspnea as a predictor of intubation are scarce. In patients receiving NIV for ARF of a mixed nature, dyspnea was associated with adverse outcomes, including NIV failure [13]. Similar results were observed in COVID-19 patients [14]. Of note, dyspnea was among intubation criteria of only one of the large randomized controlled trials published in the last decade and in which intubation was the primary or a major secondary outcome [1, 15–19]. The reason why dyspnea has been so little studied is unclear.

Here, we performed a post hoc analysis of a large scale multicenter, randomized controlled trial in which dyspnea was measured [1]. Our first objective was to quantify the prevalence and intensity of dyspnea in non-intubated patients receiving noninvasive respiratory support (either standard oxygen, HFOT or NIV delivered by an ICU ventilator) for de novo hypoxemic ARF and to examine factors associated with dyspnea. Our second objective was to investigate the association between dyspnea and intubation. Finally, we examined the association between dyspnea and mortality. Our hypotheses were that dyspnea was frequent and severe in this population, and that a more intense dyspnea was associated with a higher risk of intubation and mortality.

## Patients and methods

### Study population and design

We performed a post hoc analysis of a randomized, controlled trial (NCT 01320384, registered on 22 March 2011) conducted in 23 centers in France and Belgium [1]. In this study, 310 patients admitted to ICU with ARF were randomly assigned to receive a treatment by standard oxygen, HFOT or NIV delivered by an ICU ventilator. All patients had a respiratory rate > 25 breaths/min, a ratio of arterial oxygen partial pressure to fractional inspired oxygen (PaO_2_/FiO_2_) ≤ 300 mm Hg and a PaCO_2_ ≤ 45 mmHg. The main exclusion criteria were severe neutropenia, acute-on-chronic respiratory failure, cardiogenic pulmonary edema, shock or altered consciousness. The original parent trial was approved by ethics committees at Centre Hospitalier Universitaire de Poitiers for French study sites (n. 10.11.28, 28 December 2010) and at Cliniques Universitaires Saint-Luc, Brussels for the site in Belgium (n. 10.07.12, 3 May 2011). Written informed consent was obtained from all the patients, their next of kin or another surrogate decision-maker as appropriate. According to French law, this post hoc analysis of the original study did not need additional ethics approval as no more data were collected for this analysis. Procedures followed were in accordance with the ethical standards of the responsible committee on human experimentation and with the Helsinki Declaration of 1975, as most recently amended. Two other post hoc analyses of this study have been already published [9, 21].

### Data collection and predetermined criteria for intubation

Clinical variables, respiratory variables and blood gas samples were collected at two time points. First, at randomization in the original parent trial, during spontaneous breathing with a non-rebreathing mask, termed “baseline” in the present post hoc analysis. Second, 1 h after initiation of the allocated treatment by the randomization in the parent trial (standard oxygen, HFOT or NIV) [1]. To assess the intensity of dyspnea, patients were asked to rate their breathing discomfort (in French “*inconfort respiratoire*”) by placing a cursor on a 100 mm visual analog scale (dyspnea-VAS) bounded on the left by “no respiratory discomfort” and on the right by “worst imaginable respiratory discomfort.” Of notice, this question did not target discomfort associated with the respiratory interface. Dyspnea-VAS was used to identify four groups of patients: no dyspnea (dyspnea-VAS < 16 mm), mild dyspnea (dyspnea-VAS between 16 and 39 mm), moderate dyspnea (dyspnea-VAS between 40 and 64 mm), and severe dyspnea (dyspnea-VAS ≥ 65 mm). The 40 mm cutoff was based on the 2024 *European Respiratory Society/European Society of Intensive Care Medicine* joint statement on dyspnea in critically ill patients [11] and on the many similar features shared by dyspnea and pain (noxious sensations, common pathways, similar cortical areas involved and affective dimension) [22]. The 16 mm and 65 mm cutoffs were based on the first and third quartile of dyspnea-VAS at baseline in our cohort.

The need for invasive mechanical ventilation was defined in the original parent trial by the following prespecified criteria for endotracheal intubation: (1) signs of persisting or worsening respiratory failure, including at least two of the following criteria: respiratory rate above 40 breaths/min, lack of improvement of signs of high respiratory muscle workload, development of copious tracheal secretions, pH below 7.35, or SpO_2_ below 90% for more than 5 min; (2) hemodynamic instability; or (3) deterioration of neurologic status [1]. Of note, dyspnea was not among these criteria. Intensive care unit mortality and 90-day mortality were recorded.

### Statistical analysis

Quantitative variables were described as median (interquartile range). Qualitative variables were described as frequency (percentages). Five outcomes were analyzed: two qualitative outcomes, dyspnea at baseline and dyspnea 1 h after randomization, one quantitative outcome, the difference between dyspnea intensity at baseline and dyspnea 1 h after randomization and two censored outcomes, time from enrollment to intubation (with discharge from ICU as a competitive event) and 90-day mortality, defined as the time from enrollment to death. Patients alive at 90 days were censored at 90 days, and patients lost to follow-up were censored to their last known contact.

Factors associated with moderate-to-severe dyspnea at baseline and after the first hour were studied by multivariate logistic regression analysis. The multivariate model was built with the following variables that were considered to be clinically relevant: age, smoker, immunosuppression status, McCabe score, heart rate, systolic arterial pressure, respiratory rate, bilateral pulmonary infiltrates, PaO_2_/FiO_2_ and randomization arm in the parent trial (oxygenation strategy). Adjusted odds ratios (OR) were presented with their 95% confidence intervals (CI). Factors associated with the change in dyspnea intensity between baseline and the first hour were studied using a multivariable linear regression model, taking into account the same variables as well as dyspnea intensity at baseline.

Factors associated with time to intubation were studied using the Fine and Gray model with ICU discharge as a competing event [23]. Cumulative incidence of intubation was estimated with the Kalbfleisch and Prentice method, considering ICU discharge as a competing event [24]. These factors were compared using Gray’s test [25]. A first multivariate model was built with the following baseline variables that were considered to be clinically relevant: SAPS 2, randomization arm, bilateral pulmonary infiltrates, heart rate, systolic arterial pressure, respiratory rate, dyspnea and PaO_2_/FiO_2_ at baseline. A second multivariate model was built with the following variables that were considered to be clinically relevant 1 h after treatment initiation: heart rate, arterial pressure, respiratory rate, dyspnea and PaO_2_/FiO_2_ and change in dyspnea between baseline and 1 h after treatment initiation. Adjusted subdistribution hazard ratios (sHR) were presented with their 95% CI.

Finally, the association between dyspnea and 90-day mortality was evaluated with a Cox proportional hazard model. Cumulative incidence curves for mortality were estimated using the Kaplan–Meier estimator and compared using a log rank test. The multivariate model was built with the following variables that were considered to be clinically relevant: age, preexisting cardiac failure, immunosuppression, McCabe score, SAPS 2 on admission, SOFA score on inclusion, randomization arm, bilateral pulmonary infiltrates and dyspnea at baseline. Adjusted hazard ratios (HR) were presented with their 95% CI.

For each model, validity assumptions were checked (log-linearity and proportional hazards assumptions) and multivariate models were built using a backward selection based on AIC (Akaike information criterion) criteria.

Analyses were conducted at the two-sided α risk of 5%. No multiplicity test correction was made. All statistical analyses were performed with R statistical software, version 3.2.0 (available online at http://www.r-project.org/ and package survival, cmprsk).

## Results

### Study population and prevalence of dyspnea

Among the 313 patients included in the parent trial, three patients withdrew consent and quantification of dyspnea was missing in 51 patients at baseline and in 57 patients 1 h after treatment initiation (see Additional file 1 Figure E1). Table 1 indicates the patient characteristics at baseline.

**Table 1:** Univariate analysis: factors associated with moderate-to-severe dyspnea at baseline

	All patients (*n* = 259)	No dyspnea (*n* = 70)	Mild dyspnea (*n* = 43)	Moderate dyspnea (*n* = 84)	Severe dyspnea (*n* = 62)	*p* value
		Dyspnea-VAS < 16 mm	Dyspnea-VAS 16–39 mm	Dyspnea-VAS 40–64 mm	Dyspnea-VAS ≥ 65 mm	
Patient characteristics						
Age, years, median (IQR)	62 (48–74)	61 (46–71)	57 (45–69)	64 (48–77)	66 (53–75)	0.128
Male gender*, n* (%)	183 (71)	49 (70)	35 (81)	54 (64)	45 (73)	0.245
BMI, kg m^−2^, median (IQR)	25 (22–29)	25 (22–31)	25 (21–29)	26 (23–28)	25 (22–27)	0.407
Current or past smoking,* n* (%)	94 (36)	31 (44)	19 (44)	22 (26)	22 (35)	0.078
Preexisting cardiac failure, *n* (%)	16 (6)	6 (9)	0 (0)	6 (7)	4 (6)	0.250
Immunosuppression, *n* (%)	70 (27)	24 (34)	19 (44)	19 (23)	8 (13)	0.002
McCabe						< 0.003
1, *n* (%)	203 (78)	49 (70)	27 (63)	70 (83)	57 (92)	
2, *n* (%)	48 (18)	17 (24)	14 (33)	12 (14)	5 (8)	
3, *n* (%)	8 (3)	4 (6)	2 (5)	2 (2)	0 (0)	
SAPSII at inclusion, median (IQR)	24 (19–31)	24 (20–32)	23 (18–33)	24 (19–30)	24 (18–30)	0.948
SOFA at inclusion, median (IQR)	3 (2–5)	3 (2–4)	3 (2–5)	3 (2–5)	3 (2–5)	0.269
Cause of ARF						
Community-acquired pneumonia, *n* (%)	167 (64)	43 (61)	24 (56)	59 (70)	41 (66)	0.394
Hospital-acquired pneumonia, *n* (%)	34 (13)	6 (9)	11 (26)	11 (13)	6 (10)	0.051
Other, *n* (%)	69 (27)	24 (34)	9 (21)	17 (20)	19 (31)	0.166
At baseline, at randomization						
Respiratory rate, min^−1^, median (IQR)	31 (27–36)	30 (27–35)	30 (27–37)	32 (28–36)	33 (28–39)	0.408
Dyspnea-VAS, median (IQR)	46 (13–65)	4 (0–10)	27 (21–30)	50 (48–59)	80 (74–90)	0.001
Heart rate, min^−1^, median (IQR)	105 (93–119)	110 (92–120)	105 (92–119)	103 (92–115)	107 (96–119)	0.509
Systolic arterial pressure, mmHg, median (IQR)	121 (108–140)	120 (102–134)	120 (110–141)	121 (109–141)	122 (109–139)	0.379
Bilateral pulmonary infiltrates, *n* (%)	205 (79)	52 (74)	34 (79)	65 (77)	54 (87)	0.316
Blood gases						
PaO_2_/FiO_2_, mmHg, median (IQR)	129 (98–171)	135 (103–182)	154 (92–197)	133 (105–165)	115 (96–156)	0.231
PaCO_2_, mmHg, median (IQR)	36 (32–39)	35 (33–39)	36 (33–39)	35 (32–38)	36 (32–39)	0.829
pH, mmHg, median (IQR)	7.44 (7.41–7.47)	7.43 (7.40–7.47)	7.44 (7.41–7.46)	7.44 (7.42–7.47)	7.44 (7.40–7.46)	0.672

*VAS* visual analog scale, *IQR* interquartile range, *BMI* body mass index, *SAPS 2* simplified acute physiology score, *SOFA* sequential organ failure assessment score, *PaO*_*2*_*/FiO*_*2*_ ratio of arterial oxygen tension to inspired oxygen fraction

**Fig. 1:**
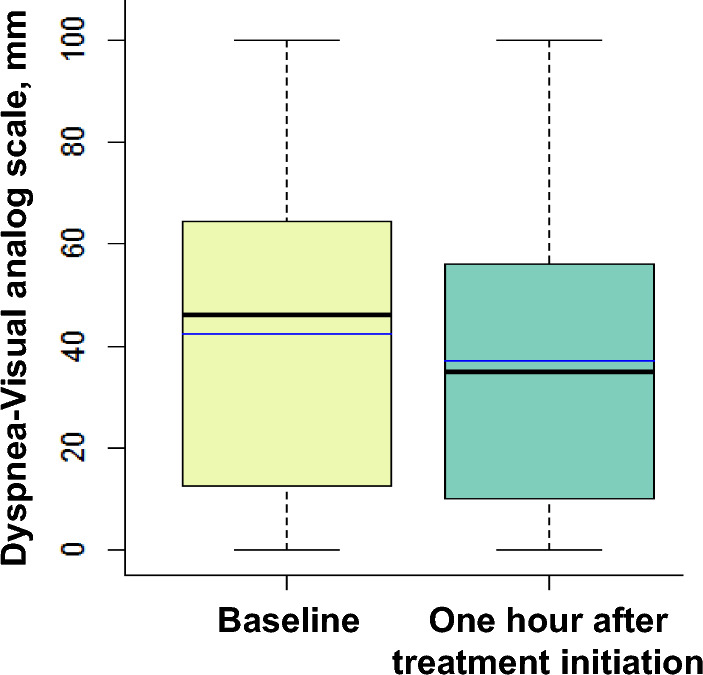
Box plots showing dyspnea-visual analog scale from zero (no respiratory discomfort) to 100 mm (worst imaginable respiratory discomfort) at baseline and 1 h after treatment initiation. The black center line denotes the median value (50th percentile), while the box contains the 25th to 75th percentiles of dataset. The blue line denotes the mean value. The black whiskers mark the maximal and minimal values

At baseline, the intensity of dyspnea was 46 (16–65) mm on the dyspnea-VAS (Fig. 1) and dyspnea was mild (dyspnea-VAS between 16 and 39 mm) in 17% of patients, moderate (dyspnea-VAS between 40 and 64 mm) in 32% and severe (dyspnea-VAS ≥ 65 mm) in 24%. After 1 h of treatment, the intensity of dyspnea score decreased to 35 (10–56) mm (*p* = 0.0004) (Fig. 1) and was mild in 22% of patients, moderate in 30% and severe in 17%. The median absolute variation of dyspnea was 10 (0–20) mm and decreased by 10 mm or more in 219 (51%) patients.

### Factors associated with moderate-to-severe dyspnea at baseline and after 1 h of treatment

Table 1 displays the factors associated with mild, moderate and severe dyspnea at baseline. On multivariate logistic regression analysis, three factors were independently associated with reduced risk of moderate-to-severe dyspnea (dyspnea-VAS ≥ 40 mm) at baseline: smoking (OR 0.39, 95% CI 0.22–0.69), immunosuppression (OR 0.48, 95% CI 0.25–0.92) and McCabe 2 or 3 (OR 0.32, 95% CI 0.16–0.65).

Table 2 shows the factors associated with mild, moderate and severe dyspnea 1 h after treatment initiation. On multivariate logistic regression analysis, six of these factors were independently associated with moderate-to-severe dyspnea (dyspnea-VAS ≥ 40 mm) 1 h after treatment initiation. Four factors were associated with increased risk of moderate-to-severe dyspnea: age > 60 years (OR 2.17, 95% CI 1.20–3.90), systolic arterial pressure 1 h after treatment initiation (OR per 10 points increased 1.13 95% CI 0.98–1.30), respiratory rate 1 h after treatment initiation (OR per 10 points increased 1.43 95% CI 0.96–2.13) and bilateral pulmonary infiltrates (OR 3.08, 95% CI 1.42–6.65). Two factors were associated with reduced risk of moderate-to-severe dyspnea: immunodeficiency (OR 0.49, 95% CI 0.25–0.97) and HFOT (OR 0.57, 95% CI 0.34–0.95).

**Table 2:** Univariate analysis: factors associated with moderate-to-severe dyspnea 1 h after treatment initiation

	All patients (*n* = 253)	No dyspnea (*n* = 80)	Mild dyspnea (*n* = 55)	Moderate dyspnea (*n* = 75)	Severe dyspnea (*n* = 43)	*p* value
		Dyspnea-VAS < 16 mm	Dyspnea-VAS 16–39 mm	Dyspnea-VAS 40–64 mm	Dyspnea-VAS ≥ 65 mm	
Patient characteristics						
Age, years, median (IQR)	60 (48–72)	56 (45–70)	59 (52–71)	62 (49–72)	69 (56–80)	0.032
Male gender, *n* (%)	178 (70)	54 (68)	42 (76)	51 (68)	31 (72)	0.676
BMI, kg m^−2^, median (IQR)	25 (22–29)	25 (22–31)	25 (22–28)	24 (22–28)	26 (23–27)	0.627
Current or past smoking, *n* (%)	94 (37)	32 (40)	19 (35)	28 (37)	15 (35)	0.911
Preexisting cardiac failure, *n* (%)	15 (6)	5 (6)	1 (2)	5 (7)	4 (9)	0.444
Immunosuppression, *n* (%)	68 (27)	29 (36)	16 (29)	17 (23)	6 (14)	0.045
McCabe						0.017
1, *n* (%)	199 (79)	54 (68)	47 (85)	62 (83)	36 (84)	
2, *n* (%)	48 (19)	23 (29)	5 (9)	13 (17)	7 (16)	
3, *n* (%)	6 (2)	3 (4)	3 (5)	0 (0)	0 (0)	
SAPSII at inclusion, median (IQR)	24 (18–30)	25 (20–31)	23 (19–29)	23 (18–30)	27 (19–32)	0.391
SOFA at inclusion, median (IQR)	3 (2–4)	3 (2–5)	3 (2–4)	3 (2–5)	3 (2–4)	0.716
Cause of ARF						
Community-acquired pneumonia, *n* (%)	168 (66)	45 (56)	42 (76)	51 (68)	30 (70)	0.092
Hospital-acquired pneumonia, *n* (%)	33 (13)	9 (11)	8 (15)	11 (15)	5 (12)	0.900
Other, *n* (%)	61 (24)	28 (35)	7 (13)	16 (21)	10 (23)	0.024
Oxygenation strategy						0.091
Standard oxygen, *n* (%)	77 (30)	22 (28)	17 (31)	24 (32)	14 (33)	
High flow oxygen therapy, *n* (%)	87 (34)	36 (45)	22 (40)	19 (25)	10 (23)	
Noninvasive ventilation, *n* (%)	89 (35)	22 (28)	16 (29)	32 (43)	19 (44)	
One hour after treatment initiation						
Respiratory rate, min^−1^, median (IQR)	29 (24–35)	29 (24–35)	27 (21–33)	30 (25–35)	31 (27–37)	0.017
Dyspnea-VAS, median (IQR)	35 (10–56)	4 (0–9)	28 (23–31)	50 (46–57)	80 (75–90)	< 0.001
Heart rate, min^−1^, median (IQR)	101 (90–115)	103 (89–117)	100 (86–109)	104 (90–116)	100 (93–118)	0.248
Systolic arterial pressure, mmHg, median (IQR)	120 (108–139)	119 (103–131)	118 (106–134)	130(113–145)	120 (107–141)	0.012
Blood gases						
PaO_2_/FiO_2_, mmHg, median (IQR)	133 (103 –173)	145 (104–187)	147 (105–179)	131 (96–165)	118 (100–157)	0.296
PaCO_2_, mmHg, median (IQR)	35 (31–39)	35 (30–38)	36 (31–40)	35 (31–38)	35 (30–40)	0.874
pH, mmHg, median (IQR)	7.44 (7.41–7.47)	7.45 (7.41–7.48)	7.44 (7.4–7.47)	7.45 (7.43–7.47)	7.43 (7.41–7.46)	0.259

*VAS* visual analog scale, *IQR* interquartile range, *BMI* body mass index, *SAPS 2* simplified acute physiology score, *SOFA* sequential organ failure assessment score, *PaO*_*2*_*/FiO*_*2*_ ratio of arterial oxygen tension to inspired oxygen fraction

On multivariable linear regression model, two factors were associated with the change in dyspnea-VAS between baseline and 1 h after treatment initiation: dyspnea-VAS at baseline (− 0.38 ± 0.05, *p* < 0.001) and noninvasive respiratory support (− 7.09 ± 3.35 for HFOT and 2.56 ± 3.36 for noninvasive ventilation as compared to standard oxygen, *p* = 0.011) (Additional file 1, Table E1). There was no difference between treatment groups at baseline in terms of dyspnea intensity. However, 1 h after treatment initiation, dyspnea-VAS decreased in the HFOT group, while it did not change in the two other groups (Additional file 1, Table E2).

### Association between dyspnea and intubation

This analysis excluded three do-not-intubate patients who died without being intubated. The intubation rate was 45% (*n* = 115).

**Fig. 2:**
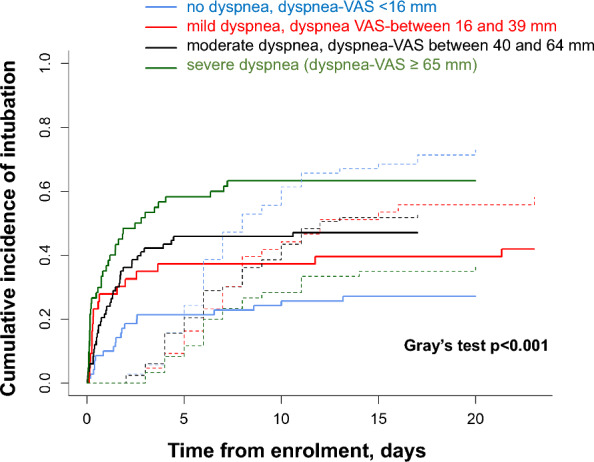
Cumulative incidence of intubation (solid line) while accounting for intensive care unit discharge (dashed line) among patients with no dyspnea at baseline (blue line), mild dyspnea (red line), moderate dyspnea (black line) and severe dyspnea (green line)

Table 3 displays the baseline variables associated with intubation while accounting for ICU discharge as a time-dependent competing risk. On Fine and Gray’s multivariate regression analysis, four variables were independently associated with intubation (Table 3). Three variables were associated with a higher risk of intubation: moderate (dyspnea-VAS 40–64 mm) and severe (dyspnea-VAS ≥ 65 mm) dyspnea at baseline (sHR 1.96, 95% CI 1.07–3.57 and sHR 2.61, 95% CI 1.40–4.87), baseline systolic arterial pressure 120–140 mmHg (sHR 2.56, 95% CI 1.58–4.17) and heart rate > 100 beat/min at baseline (sHR 1.94, 95% CI 1.29–2.92). One variable, baseline PaO_2_/FiO_2_ > 200 mmHg (sHR 0.34, 95% CI 0.15–0.76), was associated with a lower risk of intubation. The cumulative incidence of intubation was higher in patients with moderate-to-severe dyspnea at baseline than in those with no or mild dyspnea (*p* = 0.0004) (Fig. 2). There was no significant interaction between dyspnea and the randomization group (*p* = 0.071).

**Table 3:** Univariate analysis: factors associated with intubation at baseline (n = 256)

	Univariate analysis		Multivariate analysis	
	Subdistribution hazard ratio (95% confidence interval)	*p* value	Subdistribution hazard ratio (95% confidence interval)	*p* value
Patient characteristics				
Age > 60 years	1.49 (1.04–2.14)	0.030		
Gender male	1.22 (0.81–1.82)	0.345		
BMI		0.672		
25–30 kg m^−2^	1.02 (0.67–1.54)			
> 30 kg m^−2^	0.82 (0.51–1.32)			
Current or past smoking	1.16 (0.81–1.67)	0.428		
Preexisting cardiac failure	1.02 (0.52–2.02)	0.957		
Immunosuppression	0.92 (0.64–1.34)	0.679		
McCabe 2 or 3	0.79 (0.52–1.21)	0.284		
SAPS II > 25	1.46 (1.02–2.10)	0.038		
SOFA at inclusion, per 10 points	1.02 (0.93–1.13)	0.621		
Bilateral pulmonary infiltrates	1.73 (1.05–2.85)	0.032		
Cause of ARF				
Community-acquired pneumonia	0.82 (0.57–1.17)	0.268		
Hospital-acquired pneumonia	1.27 (0.76–2.13)	0.365		
Other	1.17 (0.81–1.71)	0.402		
At baseline, at randomization				
Respiratory rate ≥ 30 min^−1^	1.49 (1.03–2.17)	0.035		
Dyspnea-VAS		< 0.001		0.023
16–39 mm	1.81 (0.95–3.45)		1.54 (0.76–3.12)	
40–64 mm	2.04 (1.19–3.48)		1.96 (1.07–3.57)	
≥ 65 mm	3.31 (1.92–5.68)		2.61 (1.40–4.87)	
Heart rate ≥ 100 beat min^−1^	1.72 (1.16–2.55)	0.007	1.94 (1.29–2.92)	0.002
Systolic arterial pressure		< 0.001		< 0.001
120–140 mmHg	2.21 (1.47–3.33)		2.56 (1.58–4.17)	
> 140 mmHg	1.55 (0.96–2.51)		1.59 (0.94–2.71)	
Blood gases				
PaO_2_/FiO_2_		0.043		0.031
100–200 mmHg	0.90 (0.58–1.40)		0.77 (0.47–1.24)	
> 200 mmHg	0.43 (0.22–0.86)		0.34 (0.15–0.76)	
PaCO_2_ > 35 mmHg	0.99 (0.69–1.41)	0.942		
pH ≥ 7.40	1.07 (0.65–1.77)	0.787		
Noninvasive respiratory support (randomization arm)		0.067		0.132
Standard oxygen therapy	1		1	
High flow oxygen therapy	0.60 (0.38–0.95)		0.61 (0.36–1.04)	
Noninvasive ventilation	0.92 (0.60–1.40)		0.95 (0.58–1.56)	

The following variables were included in the initial complete model: SAPS 2, randomization arm, bilateral pulmonary infiltrates and heart rate, systolic arterial pressure, respiratory rate, dyspnea and PaO_2_/FiO_2_ at baseline

The Area Under the Curve (AUC, 95% confidence interval) of the Fine and Gray model was 76 (70–82)

*BMI* body mass index, *SAPS 2* simplified acute physiology score, *SOFA* sequential organ failure assessment score, *VAS* visual analog scale, *PaO*_*2*_*/FiO*_*2*_ ratio of arterial oxygen tension to inspired oxygen fraction

The variables measured 1 h after treatment initiation that were associated with a higher risk of intubation on Fine and Gray’s multivariate regression analysis were severe dyspnea (dyspnea-VAS ≥ 65 mm), high respiratory rate, high arterial blood pressure, high heart rate. A high PaO_2_/FiO_2_ 1 h after treatment initiation was associated with a lower risk of intubation (Additional file 1, Table E3).

### Associations between dyspnea at baseline and mortality

**Fig. 3:**
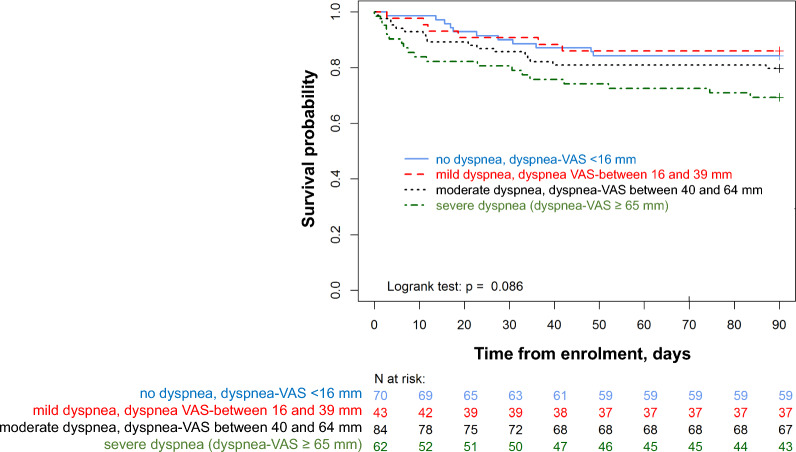
Cumulative survival up to 90 days in patients with no dyspnea at baseline (blue line), mild dyspnea (red line), moderate dyspnea (black line) and severe dyspnea (green line)

Intensive care unit mortality was 18% (*n* = 46), and 90-day mortality was 20% (*n* = 53). Ninety-day mortality was 16% (*n* = 11) in patients with no dyspnea, 16% (*n* = 6) in patients with mild dyspnea, 20% (*n* = 17) in patients with moderate dyspnea and 31% (*n* = 19) in patients with severe dyspnea (*p* = 0.110). Figure 3 shows the cumulative probability of survival up to 90 days in patients with no, mild, moderate and severe dyspnea (logrank test, *p* = 0.086). The cumulative probability of survival was lower in patients with baseline dyspnea-VAS ≥ 40 mm (logrank test, *p* = 0.049) (see Additional file 1, Figure E2).

On the multivariate Cox proportional hazard model, three factors remained in the final model and were associated with an increased 90-day mortality: SAPS 2 ≥ 25, moderate-to-severe dyspnea at baseline, PaO_2_/FiO_2_ and treatment arm (Table 4).

**Table 4:** Factors associated with 90-day mortality (n = 259)

	Univariate analysis		Multivariate analysis	
	Hazard ratio (95% confidence interval)	*p* value	Hazard ratio 95% (confidence interval)	*p* value
Patient characteristics				
Age > 60 years	2.45 (1.39–4.32)	0.001		
Gender male	0.99 (0.56–1.75)	0.979		
BMI		0.945		
25–30 kg m^−2^	0.97 (0.53–1.77)			
> 30 kg m^−2^	1.09 (9.56–2.13)			
Current or past smoking	0.71 (0.40–1.26)	0.240		
Preexisting cardiac failure	2.03 (0.87–4.72)	0.095		
Immunosuppression	1.32 (0.76–2.28)	0.327		
McCabe 2 or 3	1.59 (0.90–2.80)	0.108		
SAPS II ≥ 25	3.56 (1.95–6.51)	< 0.001	3.27 (1.68–6.35)	< 0.001
SOFA at inclusion, per point	1.16 (1.03–1.31)	0.016		
Bilateral pulmonary infiltrates	2.03 (0.92–4.49)	0.072		
Cause of ARF				
Community-acquired pneumonia	0.67 (0.40–1.14)	0.135		
Hospital-acquired pneumonia	1.50 (0.76–2.97)	0.242		
Other	1.45 (0.84–2.51)	0.184		
At baseline, at randomization				
Respiratory rate > 30 min^−1^	1.49 (0.84–2.63)	0.165		
Dyspnea-VAS		0.086		
16–39 mm	0.89 (0.33–2.41)			
40–64 mm	1.35 (0.63–2.89)			
≥ 65 mm	2.20 (1.05–4.62)			
Moderate-to-severe dyspnea^a^	1.77 (0.99–3.15)	0.049	1.73 (0.93–3.19)	0.073
Heart rate > 100 beat min^−1^	1.29 (0.74–2.25)	0.373		
Systolic arterial pressure		0.034		
120–140 mmHg	2.24 (1.19–4.20)			
> 140 mmHg	1.69 (0.86–3.33)			
Blood gases				
PaO_2_/FiO_2_		0.056		0.118
100–199 mmHg	1.32 (.069–2.53)		1.27 (0.65–2.46)	
> 200 mmHg	0.35 (1.10–1.25)		0.36 (0.08–1.64)	
PaCO_2_ > *35 mmHg*	0.93(0.55–1.57)	0.787		
pH ≥ 7.40	0.75 (0.40–1.42)	0.379		
Noninvasive respiratory support (randomization arm)		0.042		0.046
Standard oxygen therapy	1		1	
High flow oxygen therapy	0.48 (0.23–0.99)		0.43 (0.19–1.01)	
Noninvasive ventilation	1.13 (0.63–2.02)		1.08 (0.58–2.04)	

The following variables were included in the initial complete model: age, preexisting cardiac failure, immunosuppression, McCabe score, SAPS 2 on admission, SOFA score on inclusion, randomization arm, bilateral pulmonary infiltrates and dyspnea at baseline

*BMI* body mass index, *SAPS 2* simplified acute physiology score, *SOFA* sequential organ failure assessment score, *VAS* visual analog scale, *PaO*_*2*_*/FiO*_*2*_ ratio of arterial oxygen tension to inspired oxygen fraction

^a^Moderate-to severe dyspnea is defined as a dyspnea intensity ≥ 40 mm on a visual analog scale from 0 to 100 mm

## Discussion

In this ancillary study of patients admitted to the ICU for acute de novo hypoxemic ARF and treated either by noninvasive respiratory support, (1) the intensity of dyspnea at baseline was high, (2) moderate and severe dyspnea at baseline were independently associated with the risk of intubation, (3) moderate-to-severe dyspnea at baseline was also associated with 90-day mortality.

To the best of our knowledge, this is the largest study to investigate dyspnea in a population of non-intubated patients admitted for de novo hypoxemic ARF. Dyspnea has been measured as a secondary outcome in many trials [26–30] and has also been measured in a large heterogeneous population of patients receiving NIV [13]. However, the prevalence of dyspnea and its risk factors and prognostic impact have not been previously studied in a large and homogeneous population.

A major finding of our study is that poor respiratory comfort was independently associated with intubation, which has been previously reported in patients receiving NIV for ARF, for COVID-19, and in those with chronic obstructive pulmonary disease exacerbation [13, 14, 31]. This may be considered as an obvious finding, since the intensity of dyspnea is a marker of ARF severity and subsequently a symptom that physicians integrate into their intubation decision-making process. However, as opposed to signs of respiratory distress that are observed by the physician [32], dyspnea is a symptom that can only be self-reported by the patient. No study has scientifically evaluated this hypothesis, and we are not aware of physicians clearly asking their patients whether they are dyspneic or not when intubation is considered. In addition, in our study, dyspnea per se was not one of the prespecified criteria of intubation in the parent trial [1]. Subsequently, dyspnea was not supposed to be taken into account by the physician when she or he decided to intubate the patient. Dyspnea was also associated with higher mortality in our study, reinforcing the belief that dyspnea is a proxy for the severity of ARF [33]. This is in line with previous studies showing an association between poor respiratory comfort and hospital mortality in patients with suspected acute myocardial infarction [34, 35], in those admitted for acute COPD exacerbation [36] and even in patients without previously diagnosed cardiopulmonary diseases [37–39]. Dyspnea is associated with a higher intubation rate in COVID-19 patients [14], and dyspnea upon hospital discharge is also associated with higher risk of readmission or death [40, 41].

Our study highlighted the high intensity of dyspnea in patients managed for ARF in the ICU, with around half of patients reporting dyspnea of an intensity ≥ 40 mm. Similar pain intensity corresponds to the three most painful procedures experienced by ICU patients [42], and these patients should receive analgesia promptly [22]. Dyspnea can be considered as a key feature of ARF, and therefore, it is not surprising to observe such a high level of dyspnea in these patients. However, it should deserve consideration and should be managed and be controlled rapidly, like pain, to mitigate the immediate suffering and anxiety that are strongly associated with dyspnea [13, 43]. Indeed, dyspnea rated as ≥ 30/100 mm is considered to be unacceptable by one third of patients [44]. Moreover, dyspnea is involved in the dark recollections of patients following their ICU stay [45, 46] and contributes to the pathogenesis of post-traumatic stress disorders [47]. Without doubt, the relief of dyspnea is currently considered by some authors to be a basic human right [48, 49]. Educational actions are needed to ensure that identification and management of dyspnea are performed as routinely as for pain. A previous study has shown that detection of moderate-to-severe dyspnea by nurses was not followed by any therapeutic intervention, in contrast to the detection of pain, which was significantly associated with the administration of opioids [50]. There is also an urgent need to develop and validate clinical approaches to relieve dyspnea independently from the correction of its cause, exactly as in the case of pain [49].

The major strengths of our study are the prospective collection of data, especially for dyspnea level, the prespecified intubation criteria that did not include dyspnea and the multicenter design. This study presents limitations that need to be acknowledged. First, we quantified respiratory comfort at only two time points, at baseline and after 1 h. A longitudinal analysis based on multiple repeated measurements could provide additional results [31]. However, a substantial proportion of patients were intubated in the first hours of management, which causes a significant drop-off. Second, patients were not systematically assessed for delirium, which may affect the self-reporting of dyspnea. However, dyspnea was not collected in patients who were unable to provide clear and coherent answers. Third, patients were asked to quote their breathing discomfort, which is not exactly dyspnea. Although terms such as “feeling breathlessness,” “shortness of breath” or “troubles breathing,” and “getting enough air” could have been used, none of them reflects perfectly dyspnea and a recent statement recognizes “breathing discomfort” as a proxy for dyspnea [11].

*In conclusion*, this study showed that dyspnea is frequent and of a high intensity in a large proportion of critically ill patients with de novo hypoxemic ARF. Moderate-to-severe dyspnea is associated with poor outcomes and seems to be a threatening signal in these patients. As this symptom is easy to identify at the bedside, dyspnea could become a variable that is measured on a regular basis, like respiratory rate and pain intensity. Future studies are needed to evaluate the benefit of this systematic measurement on patient management and post ICU burden. Future studies should also develop strategies to relieve dyspnea, exactly as in the case of pain.

## Supplementary Information

**Additional file 1**. Supplementary figures and tables.

## Data Availability

The datasets used and/or analyzed during the current study are available from the corresponding author on reasonable request.

## Abbreviations

ICU: Intensive care unit
ARF: Acute respiratory failure
NIV: Noninvasive ventilation
HFOT: High-flow oxygen therapy
PaO_2_/FiO_2_: Ratio of arterial oxygen partial pressure to fractional inspired oxygen
SpO_2_: Pulse oxygen saturation
VAS: Visual analog scale
OR: Odds ratios
CI: 95% Confidence intervals
SHR: Subdistribution hazard ratios

## References

[1] Frat J-P, Thille AW, Mercat A, Girault C, Ragot S, Perbet S, et al. High-flow oxygen through nasal cannula in acute hypoxemic respiratory failure. N Engl J Med. 2015;372:2185–96. 25981908 10.1056/NEJMoa1503326

[2] L’Her E, Deye N, Lellouche F, Taille S, Demoule A, Fraticelli A, et al. Physiologic effects of noninvasive ventilation during acute lung injury. Am J Respir Crit Care Med. 2005;172:1112–8. 16081548 10.1164/rccm.200402-226OC

[3] Demoule A, Chevret S, Carlucci A, Kouatchet A, Jaber S, Meziani F, et al. Changing use of noninvasive ventilation in critically ill patients: trends over 15 years in francophone countries. Intensive Care Med. 2016;42:82–92. 26464393 10.1007/s00134-015-4087-4

[4] Carrillo A, Gonzalez-Diaz G, Ferrer M, Martinez-Quintana ME, Lopez-Martinez A, Llamas N, et al. Non-invasive ventilation in community-acquired pneumonia and severe acute respiratory failure. Intensive Care Med. 2012;38:458–66. 22318634 10.1007/s00134-012-2475-6

[5] Thille AW, Contou D, Fragnoli C, Cordoba-Izquierdo A, Boissier F, Brun-Buisson C. Non-invasive ventilation for acute hypoxemic respiratory failure: intubation rate and risk factors. Crit Care. 2013;17:R269. 24215648 10.1186/cc13103 PMC4057073

[6] Bellani G, Laffey JG, Pham T, Madotto F, Fan E, Brochard L, et al. Noninvasive ventilation of patients with acute respiratory distress syndrome. Insights from the LUNG SAFE study. Am J Respir Crit Care Med. 2017;195:67–77. 27753501 10.1164/rccm.201606-1306OC

[7] Antonelli M, Conti G, Moro ML, Esquinas A, Gonzalez-Diaz G, Confalonieri M, et al. Predictors of failure of noninvasive positive pressure ventilation in patients with acute hypoxemic respiratory failure: a multi-center study. Intensive Care Med. 2001;27:1718–28. 11810114 10.1007/s00134-001-1114-4

[8] Roca O, Caralt B, Messika J, Samper M, Sztrymf B, Hernández G, et al. An index combining respiratory rate and oxygenation to predict outcome of nasal high-flow therapy. Am J Respir Crit Care Med. 2019;199:1368–76. 30576221 10.1164/rccm.201803-0589OC

[9] Frat J-P, Ragot S, Coudroy R, Constantin J-M, Girault C, Prat G, et al. Predictors of intubation in patients with acute hypoxemic respiratory failure treated with a noninvasive oxygenation strategy. Crit Care Med. 2018;46:208–15. 29099420 10.1097/CCM.0000000000002818

[10] Carteaux G, Millán-Guilarte T, De Prost N, Razazi K, Abid S, Thille AW, et al. Failure of noninvasive ventilation for de novo acute hypoxemic respiratory failure: role of tidal volume. Crit Care Med. 2016;44:282–90. 26584191 10.1097/CCM.0000000000001379

[11] Demoule A, Decavele M, Antonelli M, Camporota L, Abroug F, Adler D, et al. Dyspnoea in acutely ill mechanically ventilated adult patients: an ERS/ESICM statement. Intensive Care Med. 2024;50:159–80. 38388984 10.1007/s00134-023-07246-x

[12] Schmidt M, Kindler F, Gottfried SB, Raux M, Hug F, Similowski T, et al. Dyspnea and surface inspiratory electromyograms in mechanically ventilated patients. Intensive Care Med. 2013;39:1368–76. 23575612 10.1007/s00134-013-2910-3

[13] Dangers L, Montlahuc C, Kouatchet A, Jaber S, Meziani F, Perbet S, et al. Dyspnoea in patients receiving noninvasive ventilation for acute respiratory failure: prevalence, risk factors and prognostic impact: a prospective observational study. Eur Respir J. 2018;52:1702637. 29976650 10.1183/13993003.02637-2017

[14] Menga LS, Grieco DL, Rosà T, Cesarano M, Delle Cese L, Berardi C, et al. Dyspnoea and clinical outcome in critically ill patients receiving noninvasive support for COVID-19 respiratory failure: post hoc analysis of a randomised clinical trial. ERJ Open Res. 2021;7:00418–2021. 34611526 10.1183/23120541.00418-2021 PMC8381256

[15] Frat J-P, Quenot J-P, Badie J, Coudroy R, Guitton C, Ehrmann S, et al. Effect of high-flow nasal cannula oxygen vs standard oxygen therapy on mortality in patients with respiratory failure due to COVID-19: the SOHO-COVID randomized clinical trial. JAMA. 2022;328:1212–22. 36166027 10.1001/jama.2022.15613 PMC9516287

[16] Azoulay E, Lemiale V, Mokart D, Nseir S, Argaud L, Pène F, et al. Effect of high-flow nasal oxygen vs standard oxygen on 28-day mortality in immunocompromised patients with acute respiratory failure: the HIGH randomized clinical trial. JAMA. 2018;320:2099–107. 30357270 10.1001/jama.2018.14282 PMC6583581

[17] Lemiale V, Mokart D, Resche-Rigon M, Pène F, Mayaux J, Faucher E, et al. Effect of noninvasive ventilation vs oxygen therapy on mortality among immunocompromised patients with acute respiratory failure: a randomized clinical trial. JAMA. 2015;314:1711–9. 26444879 10.1001/jama.2015.12402

[18] Ospina-Tascón GA, Calderón-Tapia LE, García AF, Zarama V, Gómez-Álvarez F, Álvarez-Saa T, et al. Effect of high-flow oxygen therapy vs conventional oxygen therapy on invasive mechanical ventilation and clinical recovery in patients with severe COVID-19: a randomized clinical trial. JAMA. 2021;326:2161–71. 34874419 10.1001/jama.2021.20714 PMC8652598

[19] Grieco DL, Menga LS, Cesarano M, Rosà T, Spadaro S, Bitondo MM, et al. Effect of helmet noninvasive ventilation vs high-flow nasal oxygen on days free of respiratory support in patients with COVID-19 and moderate to severe hypoxemic respiratory failure: the HENIVOT randomized clinical trial. JAMA. 2021;325:1731–43. 33764378 10.1001/jama.2021.4682 PMC7995134

[20] Parshall MB, Schwartzstein RM, Adams L, Banzett RB, Manning HL, Bourbeau J, et al. An official American Thoracic Society statement: update on the mechanisms, assessment, and management of dyspnea. Am J Respir Crit Care Med. 2012;185:435–52. 22336677 10.1164/rccm.201111-2042ST PMC5448624

[21] Frat J-P, Ragot S, Girault C, Perbet S, Prat G, Boulain T, et al. Effect of non-invasive oxygenation strategies in immunocompromised patients with severe acute respiratory failure: a post-hoc analysis of a randomised trial. Lancet Respir Med. 2016;4:646–52. 27245914 10.1016/S2213-2600(16)30093-5

[22] Barr J, Fraser GL, Puntillo K, Ely EW, Gélinas C, Dasta JF, et al. Clinical practice guidelines for the management of pain, agitation, and delirium in adult patients in the intensive care unit. Crit Care Med. 2013;41:263–306. 23269131 10.1097/CCM.0b013e3182783b72

[23] Fine JP, Gray RJ. A proportional hazards model for the subdistribution of a competing risk. J Am Stat Assoc. 1999;94:496–509.

[24] Kalbfleisch JD, Prentice RL. The statistical analysis of failure time data: Kalbfleisch/The statistical [Internet]. Wiley, Hoboken, NJ, USA; 2002 [cited 2022 Mar 1]. 10.1002/9781118032985.

[25] Gray RJ. A class of $K$-sample tests for comparing the cumulative incidence of a competing risk. Ann Stat. 1988. 10.1214/aos/1176350951.full.

[26] Bott J, Carroll MP, Conway JH, Keilty SE, Ward EM, Brown AM, et al. Randomised controlled trial of nasal ventilation in acute ventilatory failure due to chronic obstructive airways disease. Lancet Lond Engl. 1993;341:1555–7. 10.1016/0140-6736(93)90696-e 8099639

[27] Kramer N, Meyer TJ, Meharg J, Cece RD, Hill NS. Randomized, prospective trial of noninvasive positive pressure ventilation in acute respiratory failure. Am J Respir Crit Care Med. 1995;151:1799–806. 7767523 10.1164/ajrccm.151.6.7767523

[28] Mehta S, Jay GD, Woolard RH, Hipona RA, Connolly EM, Cimini DM, et al. Randomized, prospective trial of bilevel versus continuous positive airway pressure in acute pulmonary edema. Crit Care Med. 1997;25:620–8. 9142026 10.1097/00003246-199704000-00011

[29] Liesching T, Nelson DL, Cormier KL, Sucov A, Short K, Warburton R, et al. Randomized trial of bilevel versus continuous positive airway pressure for acute pulmonary edema. J Emerg Med. 2014;46:130–40. 24071031 10.1016/j.jemermed.2013.08.015

[30] Nava S, Ferrer M, Esquinas A, Scala R, Groff P, Cosentini R, et al. Palliative use of non-invasive ventilation in end-of-life patients with solid tumours: a randomised feasibility trial. Lancet Oncol. 2013;14:219–27. 23406914 10.1016/S1470-2045(13)70009-3

[31] Kocks JWH, van den Berg JWK, Kerstjens HAM, Uil SM, Vonk JM, de Jong YP, et al. Day-to-day measurement of patient-reported outcomes in exacerbations of chronic obstructive pulmonary disease. Int J Chronic Obstr Pulm Dis. 2013;8:273–86. 10.2147/COPD.S43992 PMC3678711 23766644

[32] Tulaimat A, Gueret RM, Wisniewski MF, Samuel J. Association between rating of respiratory distress and vital signs, severity of illness, intubation, and mortality in acutely ill subjects. Respir Care. 2014;59:1338–44. 24847098 10.4187/respcare.02650

[33] Pesola GR, Ahsan H. Dyspnea as an independent predictor of mortality. Clin Respir J. 2016;10:142–52. 25070878 10.1111/crj.12191 PMC4309743

[34] Bøtker MT, Stengaard C, Andersen MS, Søndergaard HM, Dodt KK, Niemann T, et al. Dyspnea, a high-risk symptom in patients suspected of myocardial infarction in the ambulance? A population-based follow-up study. Scand J Trauma Resusc Emerg Med. 2016;24:15. 26872739 10.1186/s13049-016-0204-9 PMC4751637

[35] Kirchberger I, Heier M, Kuch B, von Scheidt W, Meisinger C. Presenting symptoms of myocardial infarction predict short- and long-term mortality: the MONICA/KORA Myocardial Infarction Registry. Am Heart J. 2012;164:856–61. 23194485 10.1016/j.ahj.2012.06.026

[36] Steer J, Norman EM, Afolabi OA, Gibson GJ, Bourke SC. Dyspnoea severity and pneumonia as predictors of in-hospital mortality and early readmission in acute exacerbations of COPD. Thorax. 2012;67:117–21. 21896712 10.1136/thoraxjnl-2011-200332

[37] Santos M, Kitzman DW, Matsushita K, Loehr L, Sueta CA, Shah AM. Prognostic importance of dyspnea for cardiovascular outcomes and mortality in persons without prevalent cardiopulmonary disease: the atherosclerosis risk in communities study. PLoS ONE. 2016;11: e0165111. 27780208 10.1371/journal.pone.0165111 PMC5079579

[38] Frostad A, Søyseth V, Andersen A, Gulsvik A. Respiratory symptoms as predictors of all-cause mortality in an urban community: a 30-year follow-up. J Intern Med. 2006;259:520–9. 16629856 10.1111/j.1365-2796.2006.01631.x

[39] Stevens JP, Dechen T, Schwartzstein RM, O’Donnell C, Baker K, Banzett RB. Association of dyspnea, mortality, and resource use in hospitalised patients. Eur Respir J. 2021;58(3):1902107. 33653806 10.1183/13993003.02107-2019

[40] Dupuis-Lozeron E, Soccal PM, Janssens J-P, Similowski T, Adler D. Severe dyspnea is an independent predictor of readmission or death in COPD patients surviving acute hypercapnic respiratory failure in the ICU. Front Med. 2018;5:163. 10.3389/fmed.2018.00163 PMC5987049 29896476

[41] Decavèle M, Similowski T. Dyspnoea upon hospital admission: listen to the bird of ill omen! Eur Respir J. 2021;58:2100988. 34475114 10.1183/13993003.00988-2021

[42] Puntillo KA, Max A, Timsit J-F, Vignoud L, Chanques G, Robleda G, et al. Determinants of procedural pain intensity in the intensive care unit. The Europain® study. Am J Respir Crit Care Med. 2014;189:39–47. 24262016 10.1164/rccm.201306-1174OC

[43] Schmidt M, Demoule A, Polito A, Porchet R, Aboab J, Siami S, et al. Dyspnea in mechanically ventilated critically ill patients. Crit Care Med. 2011;39:2059–65. 21572329 10.1097/CCM.0b013e31821e8779

[44] Stevens JP, Sheridan AR, Bernstein HB, Baker K, Lansing RW, Schwartzstein RM, et al. A multidimensional profile of dyspnea in hospitalized patients. Chest. 2019;156:507–17. 31128117 10.1016/j.chest.2019.04.128 PMC7090324

[45] Rotondi AJ, Chelluri L, Sirio C, Mendelsohn A, Schulz R, Belle S, et al. Patients’ recollections of stressful experiences while receiving prolonged mechanical ventilation in an intensive care unit. Crit Care Med. 2002;30:746–52. 11940739 10.1097/00003246-200204000-00004

[46] de Miranda S, Pochard F, Chaize M, Megarbane B, Cuvelier A, Bele N, et al. Postintensive care unit psychological burden in patients with chronic obstructive pulmonary disease and informal caregivers: a multicenter study. Crit Care Med. 2011;39:112–8. 21037472 10.1097/CCM.0b013e3181feb824

[47] Demoule A, Hajage D, Messika J, Jaber S, Diallo H, Coutrot M, et al. Prevalence, intensity, and clinical impact of dyspnea in critically ill patients receiving invasive ventilation. Am J Respir Crit Care Med. 2022;205:917–26. 35061577 10.1164/rccm.202108-1857OC

[48] Currow DC, Abernethy AP, Ko DN. The active identification and management of chronic refractory breathlessness is a human right. Thorax. 2014;69:393–4. 24212892 10.1136/thoraxjnl-2013-204701

[49] Başoğlu M. Effective management of breathlessness: a review of potential human rights issues. Eur Respir J. 2017;49:1602099. 28546267 10.1183/13993003.02099-2016

[50] Gentzler ER, Derry H, Ouyang DJ, Lief L, Berlin DA, Xu CJ, et al. Underdetection and undertreatment of dyspnea in critically ill patients. Am J Respir Crit Care Med. 2019;199:1377–84. 30485121 10.1164/rccm.201805-0996OC PMC6543712

