# Correction: Dyspnea is severe and associated with a higher intubation rate in de novo acute hypoxemic respiratory failure

**DOI:** 10.1186/s13054-025-05314-w

**Published:** 2025-02-26

**Authors:** Alexandre Demoule, Amandine Baptiste, Arnaud W. Thille, Thomas Similowski, Stephanie Ragot, Gwénael Prat, Alain Mercat, Christophe Girault, Guillaume Carteaux, Thierry Boulain, Sébastien Perbet, Maxens Decavèle, Lisa Belin, Jean-Pierre Frat, from the REVA Network (Research Network in Mechanical Ventilation)

**Affiliations:** 1 INSERM, UMRS1158 Neurophysiologie Respiratoire Expérimentale et Clinique, Sorbonne Université, 75005 Paris, France; 2 Groupe Hospitalier Universitaire APHP-Sorbonne Université, Site Pitié-Salpêtrière, Service de Médecine Intensive et Réanimation (Département R3S), Hôpital Universitaire Pitié-Salpêtrière, AP-HP, 47-83 Boulevard de L’Hôpital, 75651 Paris Cedex 13, France; 3 Groupe Hospitalier Universitaire APHP-Sorbonne Université, Site Pitié-Salpêtrière, Unité de Recherche Clinique, AP-HP, Paris, France; 4 Médecine Intensive Réanimation, Centre Hospitalier Universitaire de Poitiers, Poitiers, France; 5 Centre d’Investigation Clinique 1402 ALIVE, Université de Poitiers, Poitiers, France; 6 Groupe Hospitalier Universitaire APHP-Sorbonne Université, Site Pitié-Salpêtrière, Département R3S, AP-HP, 75013 Paris, France; 7 Service de Médecine Intensive et Réanimation, CHU de Brest, Brest, France; 8 Service de Réanimation médicale et Médecine Hyperbare, Centre Hospitalier Régional Universitaire, Angers, France; 9 UNIROUEN, UR 3830, Medical Intensive Care Unit, Rouen University Hospital, Normandie University, Rouen, France; 10 Hôpitaux Universitaires Henri Mondor, Service de Médecine Intensive Réanimation, Université Paris Est Créteil, Groupe de Recherche Clinique CARMAS, AP-HP, Créteil, France; 11 Médecine Intensive Réanimation, Centre Hospitalier Universitaire d’Orléans, Orléans, France; 12 Réanimation Médico-Chirurgicale, CHU de Clermont-Ferrand, Clermont-Ferrand, France; 13 GReD, UMR/CNRS 6293, INSERM U1103, Université Clermont Auvergne, Clermont-Ferrand, France; 14 Site Pitié-Salpêtrière, Département de Santé Publique, INSERM, Institut Pierre Louis d’Epidémiologie Et de Santé Publique, AP-HP, APHP-Sorbonne Université, Paris, France

**Correction: Critical Care (2024) 28:174** 10.1186/s13054-024-04903-5

Following publication of the original article [1], the authors identified errors in Tables 1, 2 and 3.

On Table 1, the ratio of arterial oxygen tension to inspired oxygen fraction at inclusion was 135 (103–182) mmHg instead of 129 (98–171) mmHg in the No dyspnea group of patients (dyspnea visual analog scale [VAS] < 16 mmHg), 154 (92–197) mmHg instead of 135 (103–182) mmHg in the Mild dyspnea group (dyspnea VAS between 16 and 39 mm), 133 (105–165) mmHg instead of 154 (92–197) mmHg in the Moderate dyspnea group (dyspnea VAS 40 to 64 mm) and 115 (96–156) mmHg instead of 133 (105–165) mmHg in the Severe dyspnea group (dyspnea VAS > 65 mm). The *p* value was unaffected by these copy paste mistakes (*p* = 0.231). We also noticed that some values were not rounded.

On Table 2, the ratio of arterial oxygen tension to inspired oxygen fraction at inclusion was 133 (103–173) mmHg in the whole population instead of 146 (97–199) mmHg, 145 (104–187) instead of 133 (103–173) mmHg in the No dyspnea group of patients, 147 (105–179) mmHg instead of in the Mild dyspnea group, 131 (96–165) mmHg instead of 147 (105–179) mmHg in the Moderate dyspnea group and 118 (100–157) mmHg instead of 131 (96–165) mmHg in the Severe dyspnea group. The *p* value was unaffected by these copy paste mistakes (*p* = 0.296).

Finally, on Table 3 the authors have noted that two *p* values have moved from one line to another and that one Odds ratio value was misplaced.

In addition to these errors, there are some values that were not rounded, with decimals lefts.

None of these errors changes the original message and accuracy of the analysis.

The incorrect Table 1:

**Table 1:** Univariate analysis: factors associated with moderate-to-severe dyspnea at baseline

	All patients (*n* = 259)	No dyspnea (*n* = 70)	Mild dyspnea (*n* = 43)	Moderate dyspnea (*n* = 84)	Severe dyspnea (*n* = 62)	*p* value
		Dyspnea-VAS < 16 mm	Dyspnea-VAS 16–39 mm	Dyspnea-VAS 40–64 mm	Dyspnea-VAS ≥ 65 mm	
Patient characteristics						
Age, years, median (IQR)	62 (48–74)	61 (46–71)	57 (45–69)	64 (48–77)	66 (53–75)	0.128
Male gender, *n* (%)	183 (71)	49 (70)	35 (81)	54 (64)	45 (73)	0.245
BMI, kg m^−2^, median (IQR)	25 (22–29)	25 (22–31)	25 (21–29)	26 (23–28)	25 (22–27)	0.407
Current or past smoking,* n* (%)	94 (36)	31 (44)	19 (44)	22 (26)	22 (35)	0.078
Preexisting cardiac failure, *n* (%)	16 (6)	6 (8.57)	0 (0)	6 (7.14)	4 (6.45)	0.251
Immunosuppression, *n* (%)	70 (27)	24 (34)	19 (44)	19 (23)	8 (13)	0.002
McCabe						< 0.003
1, *n* (%)	203 (78)	49 (70)	27 (63)	70 (83)	57 (92)	
2, *n* (%)	48 (1)	17 (24)	14 (33)	12 (14)	5 (8)	
3, *n* (%)	8 (3)	4 (6)	2 (5)	2 (2)	0 (0)	
SAPSII at inclusion, median (IQR)	24 (19–31)	24 (20–32)	23 (18–33)	24 (19–30)	24 (18–30)	0.948
SOFA at inclusion, median (IQR)	3 (2–5)	3 (2–4)	3 (2–5)	3 (2–5)	3 (2–4)	0.269
Cause of ARF						
Community-acquired pneumonia, *n* (%)	167 (64)	43 (61)	24 (56)	59 (70)	41 (66)	0.394
Hospital-acquired pneumonia, *n* (%)	34 (13)	6 (9)	11 (26)	11 (13)	6 (10)	0.051
Other, *n* (%)	69 (27)	24 (34)	9 (21)	17 (20)	19 (31)	0.166
At baseline, at randomization						
Respiratory rate, min^−1^, median (IQR)	31 (27–36)	30 (27–35)	30 (27–37)	32 (28–36)	33 (28–38.75)	0.408
Dyspnea-VAS, median (IQR)	46 (13–65)	4 (0–10)	27 (21–30)	50 (47.75–59.25)	80 (74.25–90)	0.001
Heart rate, min^−1^, median (IQR)	105 (93–119)	110 (92–120)	105 (92.5–119)	103 (92–115)	107 (96–119)	0.509
Systolic arterial pressure, mmHg, median (IQR)	121 (108–140)	120 (102–134)	120 (110–141)	121 (109–141)	122 (109–139)	0.379
Bilateral pulmonary infiltrates, *n* (%)	205 (79)	52 (74)	34 (79)	65 (77)	54 (87)	0.316
Blood gases						
PaO_2_/FiO_2_, mmHg, median (IQR)	129 (98–171)	129 (98–171)	135 (103–182)	154 (92–197)	133 (105–165)	0.231
PaCO_2_, mmHg, median (IQR)	36 (32–39)	35 (33–39)	36 (33–39)	35 (32–38)	36 (32–39)	0.829
pH, mmHg, median (IQR)	7.44 (7.41–7.47)	7.43 (7.40–7.47)	7.44 (7.41–7.46)	7.44 (7.42–7.47)	7.44 (7.40–7.46)	0.572

*VAS* visual analog scale, *IQR* interquartile range, *BMI* body mass index, *SAPS 2* simplified acute physiology score, *SOFA* sequential organ failure assessment score, *PaO*_*2*_*/FiO*_*2*_ ratio of arterial oxygen tension to inspired oxygen fraction

The correct Table 1:

**Table 1:** Univariate analysis: factors associated with moderate-to-severe dyspnea at baseline

	All patients (*n* = 259)	No dyspnea (*n* = 70)	Mild dyspnea (*n* = 43)	Moderate dyspnea (*n* = 84)	Severe dyspnea (*n* = 62)	*p* value
		Dyspnea-VAS < 16 mm	Dyspnea-VAS 16–39 mm	Dyspnea-VAS 40–64 mm	Dyspnea-VAS ≥ 65 mm	
Patient characteristics						
Age, years, median (IQR)	62 (48–74)	61 (46–71)	57 (45–69)	64 (48–77)	66 (53– 75)	0.128
Male gender, *n* (%)	183 (71)	49 (70)	35 (81)	54 (64)	45 (73)	0.245
BMI, kg m^−2^, median (IQR)	25 (22–29)	25 (22–31)	25 (21–29)	26 (23–28)	25 (22–27)	0.407
Current or past smoking, *n* (%)	94 (36)	31 (44)	19 (44)	22 (26)	22 (35)	0.078
Pre-existing cardiac failure, *n* (%)	16 (6)	6 (9)	0 (0)	6 (7)	4 (6)	0.250
Immunosuppression, *n* (%)	70 (27)	24 (34)	19 (44)	19 (23)	8 (13)	0.002
McCabe						0.003
1, *n* (%)	203 (78)	49 (70)	27 (63)	70 (83)	57 (92)	
2, *n* (%)	48 (18)	17 (24)	14 (33)	12 (14)	5 (8)	
3, *n* (%)	8 (3)	4 (6)	2 (5)	2 (2)	0 (0)	
SAPSII at inclusion, median (IQR)	24 (19–31)	24 (20–32)	23 (18–33)	24 (19–30)	24 (18–30)	0.948
SOFA at inclusion, median (IQR)	3 (2–5)	3 (2–4)	3 (2–5)	3 (2–5)	3 (2–5)	0.269
Cause of ARF						
Community-acquired pneumonia, *n* (%)	167 (64)	43 (61)	24 (56)	59 (70)	41 (66)	0.394
Hospital-acquired pneumonia, *n* (%)	34 (13)	6 (9)	11 (26)	11 (13)	6 (10)	0.051
Other, *n* (%)	69 (27)	24 (34)	9 (21)	17 (20)	19 (31)	0.166
At baseline, at randomization						
Respiratory rate, min^−1^, median (IQR)	31 (27–36)	30 (27–35)	30 (27–37)	32 (28–36)	33 (28–39)	0.408
Dyspnea-VAS, median (IQR)	46 (13–65)	4 (0–10)	27 (21–30)	50 (48–59)	80 (74–90)	< 0.001
Heart rate, min^−1^, median (IQR)	105 (93–119)	110 (92–120)	105 (92–119)	103 (92–115)	107 (96–119)	0.509
Systolic arterial pressure, mmHg, median (IQR)	121 (108–140)	120 (102–134)	120 (110–141)	121 (109–141)	122 (109–139)	0.379
Bilateral pulmonary infiltrates, *n* (%)	205 (79)	52 (74)	34 (79)	65 (77)	54 (87)	0.316
Blood gases						
PaO_2_/FiO_2_, mmHg, median (IQR)	129 (98–171)	135 (103–182)	154 (92–197)	133 (105–165)	115 (96–156)	0.231
PaCO_2_, mmHg, median (IQR)	36 (32–39)	35 (33–39)	36 (33–39)	35 (32–38)	36 (32–39)	0.829
pH, mmHg, median (IQR)	7.44 (7.41–7.47)	7.43 (7.40–7.47)	7.44 (7.41–7.46)	7.44 (7.42–7.47)	7.44 (7.40–7.46)	0.672

*VAS* visual analog scale, *IQR* interquartile range, *BMI* body mass index, *SAPS 2* simplified acute physiology score, *SOFA* sequential organ failure assessment score, *PaO*_*2*_*/FiO*_*2*_ ratio of arterial oxygen tension to inspired oxygen fraction

The incorrect Table 2:

**Table 2:** Univariate analysis: factors associated with moderate to severe dyspnea 1 h after treatment initiation

	All patients (*n* = 253)	No dyspnea (*n* = 80)	Mild dyspnea (*n* = 55)	Moderate dyspnea (*n* = 75)	Severe dyspnea (*n* = 43)	*p* value
		Dyspnea-VAS < 16 mm	Dyspnea-VAS 16–39 mm	Dyspnea-VAS 40–64 mm	Dyspnea-VAS ≥ 65 mm	
Patient characteristics						
Age, years, median (IQR)	60 (48–72)	56 (45–70)	59 (52–71)	62 (49–72)	69 (56–80)	0.032
Male gender, *n* (%)	178 (70)	54 (68)	42 (76)	51 (68)	31 (72)	0.676
BMI, kg m^−2^, median (IQR)	25 (22–29)	25 (22–31)	25 (22–28)	24 (21.75–28)	26 (23–27)	0.627
Current or past smoking, *n* (%)	94 (37)	32 (40)	19 (35)	28 (37)	15 (35)	0.911
Preexisting cardiac failure, *n* (%)	15 (9)	5 (6)	1 (2)	5 (7)	4 (9)	0.444
Immunosuppression, *n* (%)	68 (27)	29 (36)	16 (29)	17 (23)	6 (14)	0.045
McCabe						0.017
1, *n* (%)	199 (79)	54 (68)	47 (85)	62 (83)	36 (84)	
2, *n* (%)	48 (19)	23 (29)	5 (9)	13 (17)	7 (16)	
3, *n* (%)	6 (2)	3 (4)	3 (5)	0 (0)	0 (0)	
SAPSII at inclusion, median (IQR)	24 (18–30)	25 (20–31)	23 (19–29)	23 (18–30)	27 (19–32)	0.391
SOFA at inclusion, median (IQR)	3 (2–4)	3 (2–5)	3 (2–4)	3 (2–5)	3 (2–4)	0.716
Cause of ARF						
Community-acquired pneumonia, *n* (%)	168 (66)	45 (56)	42 (76)	51 (68)	30 (70)	0.092
Hospital-acquired pneumonia, *n* (%)	33 (13)	9 (11)	8 (15)	11 (15)	5 (12)	0.900
Other, *n* (%)	63 (25)	28 (35)	7 (13)	16 (21)	10 (23)	0.024
Oxygenation strategy						0.091
Standard oxygen, *n* (%)	77 (30)	22 (28)	17 (301)	24 (32)	14 (33)	
High flow oxygen therapy, *n* (%)	87 (34)	36 (45)	22 (40)	19 (25)	10 (23)	
Noninvasive ventilation, *n* (%)	89 (35)	22 (28)	16 (29)	32 (43)	19 (44)	
One hour after treatment initiation						
Respiratory rate, min^−1^, median (IQR)	29 (24–35)	29 (24–35)	26.5 (21–33)	30 (25–35)	31 (27–37)	0.017
Dyspnea-VAS, median (IQR)	35 (10–56)	4 (0–9)	28 (23–31)	50 (46–57)	80 (75–90)	< 0.001
Heart rate, min^−1^, median (IQR)	101 (90–115)	103 (89–117)	100 (86–109)	104 (90–116)	100 (93–118)	0.248
Systolic arterial pressure, mmHg, median (IQR)	120 (108–139)	119 (103–131)	118 (106–134)	130(113–145)	120 (107–141)	0.012
Blood gases						
PaO_2_/FiO_2_, mmHg, median (IQR)	146 (97–199)	133 (103 –173)	145 (104–187)	147 (105–179)	131 (96–165)	0.296
PaCO_2_, mmHg, median (IQR)	35 (31–39)	35 (30–38)	36 (31–40)	35 (31–38)	35 (30–40)	
pH, mmHg, median (IQR)	7.44 (7.41–7.47)	7.45 (7.41–7.48)	7.44 (7.4–7.47)	7.45 (7.43–7.47)	7.43 (7.41–7.46)	0.259

*VAS* visual analog scale, *IQR* interquartile range, *BMI* body mass index, *SAPS 2* simplified acute physiology score, *SOFA* sequential organ failure assessment score, *PaO*_*2*_*/FiO*_*2*_ ratio of arterial oxygen tension to inspired oxygen fraction

The correct Table 2:

**Table 2:** Univariate analysis: factors associated with moderate to severe dyspnea 1 h after treatment initiation

	All patients (*n* = 253)	No dyspnea (*n* = 80)	Mild dyspnea (*n* = 55)	Moderate dyspnea (*n* = 75)	Severe dyspnea (*n* = 43)	*p* value
		Dyspnea-VAS < 16 mm	Dyspnea-VAS 16–39 mm	Dyspnea-VAS 40–64 mm	Dyspnea-VAS ≥ 65 mm	
Patient characteristics						
Age, years, median (IQR)	60 (48–72)	56 (45–70)	59 (52–71)	62 (49–72)	69 (56–80)	0.032
Male gender, *n* (%)	178 (70)	54 (68)	42 (76)	51 (68)	31 (72)	0.676
BMI, kg.m^−2^, median (IQR)	25 (22–29)	25 (22–31)	25 (22–28)	24 (22–28)	26 (23–27)	0.627
Current or past smoking, *n* (%)	94 (37)	32 (40)	19 (35)	28 (37)	15 (35)	0.911
Pre-existing cardiac failure, *n* (%)	15 (6)	5 (6)	1 (2)	5 (7)	4 (9)	0.444
Immunosuppression, *n* (%)	68 (27)	29 (36)	16 (29)	17 (23)	6 (14)	0.045
McCabe						0.017
1, *n* (%)	199 (79)	54 (68)	47 (85)	62 (83)	36 (84)	
2, *n* (%)	48 (19)	23 (29)	5 (9)	13 (17)	7 (16)	
3, *n* (%)	6 (2)	3 (4)	3 (5)	0 (0)	0 (0)	
SAPSII at inclusion, median (IQR)	24 (18–30)	25 (20–31)	23 (19–29)	23 (18–30)	27 (19–32)	0.391
SOFA at inclusion, median (IQR)	3 (2–4)	3 (2–5)	3 (2–4)	3 (2–5)	3 (2–4)	0.716
Cause of ARF						
Community-acquired pneumonia, *n* (%)	168 (66)	45 (56)	42 (76)	51 (68)	30 (70)	0.092
Hospital-acquired pneumonia, *n* (%)	33 (13)	9 (11)	8 (15)	11 (15)	5 (12)	0.900
Other, *n* (%)	61 (24)	28 (35)	7 (13)	16 (21)	10 (23)	0.024
Oxygenation strategy						0.091
Standard oxygen, *n* (%)	77 (30)	22 (28)	17 (31)	24 (32)	14 (33)	
High flow oxygen therapy, *n* (%)	87 (34)	36 (45)	22 (40)	19 (25)	10 (23)	
Noninvasive ventilation, *n* (%)	89 (35)	22 (28)	16 (29)	32 (43)	19 (44)	
One hour after treatment initiation						
Respiratory rate, min^−1^, median (IQR)	29 (24–35)	29 (24–35)	27 (21–33)	30 (25–35)	31 (27–37)	0.017
Dyspnea-VAS, median (IQR)	35 (10–56)	4 (0–9)	28 (23–31)	50 (46–57)	80 (75–90)	< 0.001
Heart rate, min^−1^, median (IQR)	101 (90–115)	103 (89–117)	100 (86–109)	104 (90–116)	100 (93–118)	0.248
Systolic arterial pressure, mmHg, median (IQR)	120 (108–139)	119 (103–131)	118 (106–134)	130(113–145)	120 (107–141)	0.012
Blood gases						
PaO_2_/FiO_2_, mmHg, median (IQR)	133 (103 –173)	145 (104–187)	147 (105–179)	131 (96–165)	118 (100–157)	0.296
PaCO_2_, mmHg, median (IQR)	35 (31–39)	35 (30–38)	36 (31–40)	35 (31–38)	35 (30–40)	0.874
pH, mmHg, median (IQR)	7.44 (7.41–7.47)	7.45 (7.41–7.48)	7.44 (7.4–7.47)	7.45 (7.43–7.47)	7.43 (7.41–7.46)	0.259

*VAS* visual analog scale, *IQR* interquartile range, *BMI* body mass index, *SAPS 2* simplified acute physiology score, *SOFA* sequential organ failure assessment score, *PaO*_*2*_*/FiO*_*2*_ ratio of arterial oxygen tension to inspired oxygen fraction

The incorrect Table 3:

**Table 3:** Univariate analysis: factors associated with intubation at baseline (*n* = 256)

	Univariate analysis		Multivariate analysis	
	Subdistribution hazard ratio (95% confidence interval)	*p* value	Subdistribution hazard ratio (95% confidence interval)	*p* value
Patient characteristics				
Age > 60 years	1.49 (1.04–2.14)	0.030		
Gender male	1.22 (0.81–1.82)	0.345		
BMI				
25–30 kg m^−2^	1.02 (0.67–1.54)	0.672		
> 30 kg m^−2^ Current or past smoking	0.82 (0.51–1.32) 1.16 (0.81–1.67)	0.428		
Preexisting cardiac failure	1.02 (0.52–2.02)	0.957		
Immunosuppression	0.92 (0.64–1.34)	0.679		
McCabe 2 or 3	0.79 (0.52–1.21)	0.284		
SAPS II > 25	1.46 (1.02–2.10)	0.038		
SOFA at inclusion, per 10 points	1.02 (0.93–1.13)	0.621		
Bilateral pulmonary infiltrates	1.73 (1.05–2.85)	0.032		
Cause of ARF				
Community-acquired pneumonia	0.82 (0.57–1.17)	0.268		
Hospital-acquired pneumonia	1.27 (0.76–2.13)	0.365		
Other	1.17 (0.81–1.71)	0.402		
At baseline, at randomization				
Respiratory rate > 30 min^−1^	1.49 (1.03–2.17)	0.035		
Dyspnea-VAS	1.96 (1.33–2.90)	< 0.001		0.023
16–39 mm	1.81 (0.95–3.45)		1.54 (0.76–3.12)	
40–64 mm	2.04 (1.19–3.48)		1.96 (1.07–3.57)	
≥ 65 mm	3.31 (1.92–5.68)		2.61 (1.40–4.87)	
Heart rate > 100 beat min^−1^	1.72 (1.16–2.55)	0.007	1.94 (1.29–2.92)	0.002
Systolic arterial pressure		< 0.001		< 0.001
120–140 mmHg	2.21 (1.47–3.33)		2.56 (1.58–4.17)	
> 140 mmHg	1.55 (0.96–2.51)		1.59 (0.94–2.71)	
Blood gases				
PaO_2_/FiO_2_		0.043		0.028
100–199 mmHg	0.90 (0.58–1.40)		0.77 (0.47–1.24)	
> 200 mmHg	0.43 (0.22–0.86)		0.34 (0.15–0.76)	
PaCO_2_ > 35 mmHg	0.99 (0.69–1.41)	0.942		
pH ≥ 7.40	1.07 (0.65–1.77)	0.787		
Noninvasive respiratory support (randomization arm)		0.067		0.132
Standard oxygen therapy	1		1	
High flow oxygen therapy	0.60 (0.38–0.95)		0.61 (0.36–1.04)	
Noninvasive ventilation	0.92 (0.60–1.40)		0.95 (0.58–1.56)	

The following variables were included in the initial complete model: SAPS 2, randomization arm, bilateral pulmonary infiltrates and heart rate, systolic arterial pressure, respiratory rate, dyspnea and PaO_2_/FiO_2_ at baseline

The Area Under the Curve (AUC, 95% confidence interval) of the Fine and Gray model was 76 (70–82)

*BMI* body mass index, *SAPS 2* simplified acute physiology score, *SOFA* sequential organ failure assessment score, *VAS* visual analog scale, *PaO*_*2*_*/FiO*_*2*_ ratio of arterial oxygen tension to inspired oxygen fraction

The correct Table 3:

**Table 3:** Univariate analysis: factors associated with intubation at baseline (*n* = 256)

	Univariate analysis		Multivariate analysis	
	Subdistribution hazard ratio (95% confidence interval)	*p* value	Subdistribution hazard ratio (95% confidence interval)	*p* value
Patient characteristics				
Age > 60 years	1.49 (1.04–2.14)	0.030		
Gender male	1.22 (0.81–1.82)	0.345		
BMI		0.672		
25–30 kg.m^−2^	1.02 (0.67–1.54)			
> 30 kg.m^−2^	0.82 (0.51–1.32)			
Current or past smoking	1.16 (0.81–1.67)	0.428		
Pre-existing cardiac failure	1.02 (0.52–2.02)	0.957		
Immunosuppression	0.92 (0.64–1.34)	0.679		
McCabe 2 or 3	0.79 (0.52–1.21)	0.284		
SAPS II > 25	1.46 (1.02–2.10)	0.038		
SOFA at inclusion, per 10 points	1.02 (0.93–1.13)	0.621		
Bilateral pulmonary infiltrates	1.73 (1.05–2.85)	0.032		
Cause of ARF				
Community-acquired pneumonia	0.82 (0.57–1.17)	0.268		
Hospital-acquired pneumonia	1.27 (0.76–2.13)	0.365		
Other	1.17 (0.81–1.71)	0.402		
At baseline, at randomization				
Respiratory rate ≥ 30 min^−1^	1.49 (1.03–2.17)	0.035		
Dyspnea-VAS		< 0.001		0.023
16–39 mm	1.81 (0.95–3.45)		1.54 (0.76–3.12)	
40–64 mm	2.04 (1.19–3.48)		1.96 (1.07–3.57)	
≥ 65 mm	3.31 (1.92–5.68)		2.61 (1.40–4.87)	
Heart rate ≥ 100 beat min^−1^	1.72 (1.16–2.55)	0.007	1.94 (1.29–2.92)	0.002
Systolic arterial pressure		< 0.001		< 0.001
120–140 mmHg	2.21 (1.47–3.33)		2.56 (1.58–4.17)	
> 140 mmHg	1.55 (0.96–2.51)		1.59 (0.94–2.71)	
Blood gases				
PaO_2_/FiO_2_		0.043		0.031
100–200 mmHg	0.90 (0.58–1.40)		0.77 (0.47–1.24)	
> 200 mmHg	0.43 (0.22–0.86)		0.34 (0.15–0.76)	
PaCO_2_ > 35 mmHg	0.99 (0.69–1.41)	0.942		
pH ≥ 7.40	1.07 (0.65–1.77)	0.787		
Non-invasive respiratory support (randomization arm)		0.067		0.132
Standard oxygen therapy	1		1	
High flow oxygen therapy	0.60 (0.38–0.95)		0.61 (0.36–1.04)	
Noninvasive ventilation	0.92 (0.60–1.40)		0.95 (0.58–1.56)	

The following variables were included in the initial complete model: SAPS 2, randomization arm, bilateral pulmonary infiltrates and heart rate, systolic arterial pressure, respiratory rate, dyspnea and PaO_2_/FiO_2_ at baseline

The Area Under the Curve (AUC, 95% confidence interval) of the Fine and Gray model was 76 (70–82)

*BMI* body mass index, *SAPS 2* simplified acute physiology score, *SOFA* sequential organ failure assessment score, *VAS* visual analog scale, *PaO*_*2*_*/FiO*_*2*_ ratio of arterial oxygen tension to inspired oxygen fraction

Tables 1, 2 and 3 have been updated in this correction article and the original article [1] has been corrected.
