# Supplementary material for: Dyspnea is severe and associated with a higher intubation rate in de novo acute hypoxemic respiratory failure
Source: Crit Care. 2024 May 23;28:174. doi: 10.1186/s13054-024-04903-5 (PMC11118550; doi:10.1186/s13054-024-04903-5)

Dyspnea is severe and associated with a higher intubation rate in *de novo* acute hypoxemic respiratory failure

Online Supplement

Figure E1. Study flow chart

Table E1. Factors associated with a decrease of dyspnea visual analog scale (Dyspnea-VAS) between baseline (patient randomization) and 1 hour after treatment initiation

Table E2. Factors associated with a decrease of dyspnea visual analog scale (dyspnea-VAS) between baseline (patient randomization) and 1 hour after treatment initiation (n=253)

Table E3. Univariate analysis: factors associated with intubation 1 hour after treatment initiation (n=253)

Figure E2. Cumulative incidence of survival up to 90 days in patients with moderate-to-severe dyspnea at baseline (dashed red line) and no or mild dyspnea (solid blue line)

Figure E1. Study flow chart

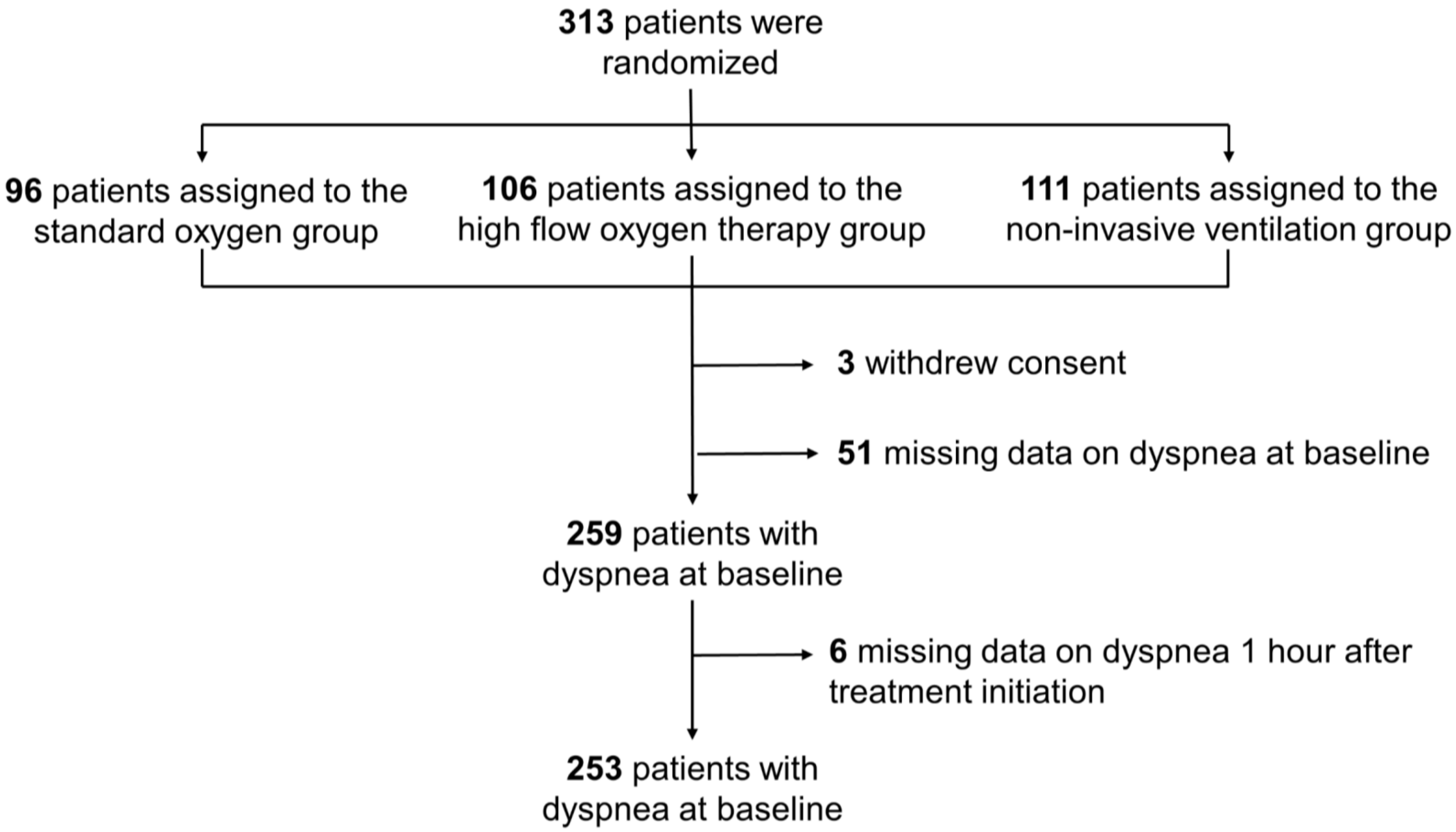

Table E1. Factors associated with a decrease of dyspnea visual analog scale (Dyspnea-VAS) between baseline (patient randomization) and 1 hour after treatment initiation

	**Univariate analysis**		**Multivariate analysis**	
	**Linear regression coefficient ± SE**	**P value**	**Estimate ± SE**	**P value**
**Patient characteristics**				
Age > 60 years	5.40 ± 3.00	0.073		
Gender *male*	3.52 ± 3.31	0.288		
BMI		0.343		
25-30 $kg.m^{-2}$	2.41 ± 3.51			
>30 $kg.m^{-2}$	5.88 ± 4.03			
Current or past smoking	3.76 ± 3.10	0.227		
Pre-existing cardiac failure	3.79 ± 6.25	0.544		
Immunosuppression	4.33 ± 3.41	0.205		
McCabe 2 or 3	5.80 ± 3.71	0.119		
SAPS II, *per 10 points*	0.27 ± 0.16	0.097		
SOFA at inclusion, *per points*	1.05 ± 0.81	0.194		
Bilateral pulmonary infiltrates	3.13 ± 3.72	0.402		
**Cause of ARF**				
Community-acquired pneumonia	-1.26 ± 3.19	0.692		
Hospital-acquired pneumonia	3.66 ± 4.45	0.412		
Other	-1.99 ± 3.51	0.571		
**At baseline, at randomization**				
Dyspnea-VAS	-0.36 ± 0.04	<0.001	-0.38 ± 0.05	<0.001
Respiratory rate, *per cycles* min^{-1}	-0.09 ± 0.24	0.711		
Heart rate, *per 10 beat* min^{-1}	-0.01 ± 0.08	0.940		
Systolic arterial pressure, *per 10 mmHg*	0.09 ± 0.07	0.187		
Blood gases				
$PaCO_2$, *per 10 mmHg*	0.16 ± 0.27	0.559		
pH, *per 0.1 point*	7.22 ± 27.07	0.790		
**Non-invasive respiratory support (randomization arm)**		0.117		0.011
Standard oxygen therapy	1		1	
High flow oxygen therapy	-6.00 ± 3.71		-7.09 ± 3.35	
Noninvasive ventilation	0.97 ± 3.70		2.56 ± 3.36	

BMI, body mass index; SAPS 2, Simplified Acute Physiology Score; SOFA, Sequential Organ Failure Assessment score; VAS, visual analog scale.

Table E2. Dyspnea-VAS at baseline, one hour after treatment initiation and the change in dyspnea-VAS between baseline and the first hour after initiation of respiratory support in the three treatment groups

**Variable**	**Standard O2**	**High flow oxygen therapy**	**Non-invasive ventilation**	**All patients**	**P value**
Dyspnea-VAS at baseline, mm	45 (18 – 634)	40 (8 – 63)	50 (20 – 70)	47 (12 – 65)	0.210
Dyspnea-VAS one hour after treatment initiation, mm	37 (15 – 58)	25 (5 – 50)	45 (22 – 61)	35 (11 – 56)	0.002
Change in dyspnea-VAS between baseline and the first hour after treatment initiation	0 (-10 – 0.5)	-6 (-22 – 0)	0 (-18 – 10)	-2 (-19 – 2)	NA
**P value, Student paired test**	0.105	<0.001	0.360	<0.001	

Table E3. Univariate analysis: factors associated with intubation 1 hour after treatment initiation (n=253)

	Univariate analysis		Multivariate analysis	
	**Subdistribution hazard ratio (95 % confidence interval)**	**P value**	**Subdistribution hazard ratio (95 % confidence interval)**	**P value**
**Patient characteristics**				
Age > 60 years	1.47 (1.02–2.11)	0.037		
Gender *male*	1.20 (0.80–1.81)	0.374		
BMI				
25-30 $kg.m^{-2}$	1.02 (0.67–1.54)	0.561		
>30 $kg.m^{-2}$	0.78 (0.48–1.27)			
Current or past smoking	1.12 (0.82–1.69)	0.428		
Pre-existing cardiac failure	1.03 (0.52–2.04)	0.938		
Immunosuppression	0.93 (0.64–1.36)	0.721		
McCabe 2 or 3	0.80 (0.52–1.23)	0.305		
SAPS II > 25	1.44 [1.01,2.07]	0.047		
SOFA at inclusion	1.03 (0.93–1.13)	0.562		
Bilateral pulmonary infiltrates	1.71 (1.04–2.83)	0.035		
**Cause of ARF**				
Community-acquired pneumonia	0.81 (0.56–1.15)	0.238		
Hospital-acquired pneumonia	1.28 (0.76–2.16)	0.346		
Other	1.19 (0.81–1.73)	0.370		
**1 hour after treatment initiation**				
Respiratory rate > 30 min^{-1}	2.02 (1.40–2.91)	<0.001	1.93 (1.21–3.09)	0.006
Dyspnea-VAS		<0.001		<0.001
16–39 mm	0.77 (0.42–1.40)		0.94 (0.52–1.70)	
40–64 mm	1.29 (0.80–2.07)		1.37 (0.80–2.36)	
≥65 mm	2.54 (1.49–4.34)		4.14 (2.04–8.41)	
Change in dyspnea intensity between baseline and 1 hour, *per 10 mm*	0.97 (0.89–1.07)	0.597	0.91 (0.83–1.01)	0.077
Heart *rate > 100 beat* min^{-1}	2.25 (1.53–3.31)	<0.001	2.43 (1.48–3.97)	<0.001
Systolic arterial pressure		0.019		0.003
120-140 mmHg	1.78 (1.13–2.79)		2.38 (1.42–3.98)	
>140 mmHg	1.86 (1.15–3.02)		1.86 (1.05–3.29)	
Blood gases				
PaO_2/FiO_2		<0.001		<0.001
100–199 mmHg	0.49 (0.32–0.76)		0.48 (0.29–0.79)	
> 200 mmHg	0.34 (0.20–0.59)		0.31 (0.16–0.60)	
$PaCO_2$ *> 35 mmHg*	1.17 (0.81–1.69)	0.414		
pH ≥ 7.40	0.58 (0.37–0.91)	0.019	0.59 (0.36–0.98)	0.041
**Non-invasive respiratory support (randomization arm)**		0.078		0.062
Standard oxygen therapy	1		1	
High flow oxygen therapy	0.61 (0.38–0.98)		0.57 (0.34–0.95)	

Noninvasive ventilation	0.94 (0.62–1.44)		0.93 (0.58–1.51)	

BMI, body mass index; SAPS 2, Simplified Acute Physiology Score; SOFA, Sequential Organ Failure Assessment score; VAS, visual analog scale; PaO_2/FiO_2, ratio of arterial oxygen tension to inspired oxygen fraction.

The following variables were included in the initial complete model: SAPS 2, randomization arm, bilateral pulmonary infiltrates and heart rate, systolic arterial pressure, respiratory rate, dyspnea and PaO_2/FiO_2 at baseline.

The Area Under the Curve (AUC, 95% confidence interval) of the Fine and Gray model was 80 (73-86).

Figure E2. Cumulative incidence of survival up to 90 days in patients with moderate-to-severe dyspnea at baseline (dashed red line) and no or mild dyspnea (solid blue line)

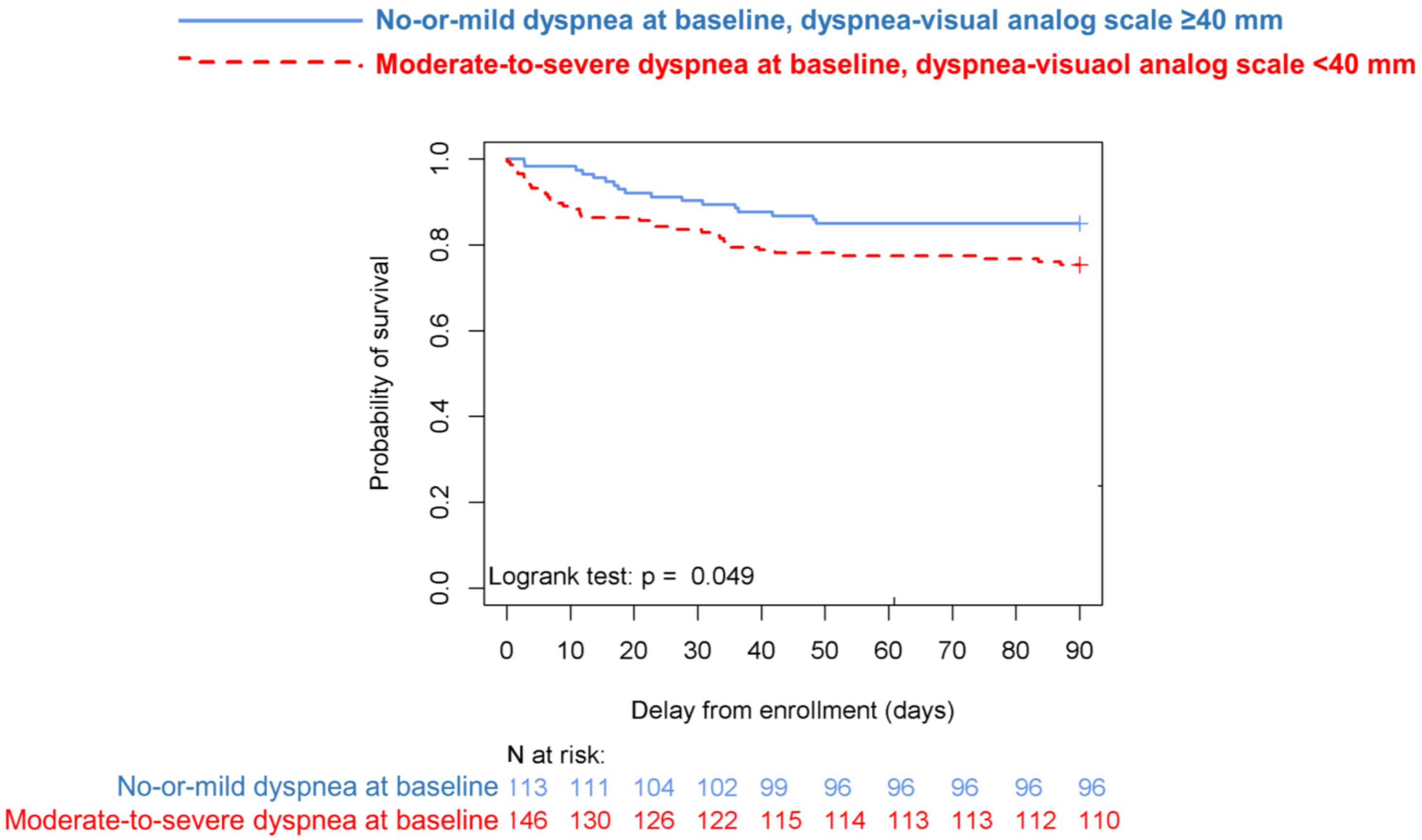

Supplement: Supplementary file 1 — Additional file 1. Supplementary figures and tables. [file 13054_2024_4903_MOESM1_ESM.pdf]
